# The Impact of Point-of-Care Testing for Influenza on Antimicrobial Stewardship (PIAMS) in UK Primary Care: Protocol for a Mixed Methods Study

**DOI:** 10.2196/46938

**Published:** 2023-06-16

**Authors:** Uy Hoang, Alice Williams, Jessica Smylie, Carole Aspden, Elizabeth Button, Jack Macartney, Cecilia Okusi, Rachel Byford, Filipa Ferreira, Meredith Leston, Charis Xuan Xie, Mark Joy, Gemma Marsden, Tristan Clark, Simon de Lusignan

**Affiliations:** 1 Nuffield Department of Primary Care Health Sciences University of Oxford Oxford United Kingdom; 2 Wolfson Institute of Population Health Barts and The London School of Medicine and Dentistry Queen Mary University of London London United Kingdom; 3 Royal College of General Practitioners London United Kingdom; 4 School of Clinical and Experimental Sciences Faculty of Medicine University of Southampton Southampton United Kingdom; 5 University Hospital Southampton National Health Service Foundation Trust Southampton United Kingdom

**Keywords:** medical records systems, computerized, influenza point-of-care systems, general practice, RSV, implementation, outcome assessment, health care, antimicrobial stewardship, acute respiratory infection, antimicrobial, influenza, primary care, respiratory symptom

## Abstract

**Background:**

Molecular point-of-care testing (POCT) used in primary care can inform whether a patient presenting with an acute respiratory infection has influenza. A confirmed clinical diagnosis, particularly early in the disease, could inform better antimicrobial stewardship. Social distancing and lockdowns during the COVID-19 pandemic have disturbed previous patterns of influenza infections in 2021. However, data from samples taken in the last quarter of 2022 suggest that influenza represents 36% of sentinel network positive virology, compared with 24% for respiratory syncytial virus. Problems with integration into the clinical workflow is a known barrier to incorporating technology into routine care.

**Objective:**

This study aims to report the impact of POCT for influenza on antimicrobial prescribing in primary care. We will additionally describe severe outcomes of infection (hospitalization and mortality) and how POCT is integrated into primary care workflows.

**Methods:**

The impact of POCT for influenza on antimicrobial stewardship (PIAMS) in UK primary care is an observational study being conducted between December 2022 and May 2023 and involving 10 practices that contribute data to the English sentinel network. Up to 1000 people who present to participating practices with respiratory symptoms will be swabbed and tested with a rapid molecular POCT analyzer in the practice. Antimicrobial prescribing and other study outcomes will be collected by linking information from the POCT analyzer with data from the patient’s computerized medical record. We will collect data on how POCT is incorporated into practice using data flow diagrams, unified modeling language use case diagrams, and Business Process Modeling Notation.

**Results:**

We will present the crude and adjusted odds of antimicrobial prescribing (all antibiotics and antivirals) given a POCT diagnosis of influenza, stratifying by whether individuals have a respiratory or other relevant diagnosis (eg, bronchiectasis). We will also present the rates of hospital referrals and deaths related to influenza infection in PIAMS study practices compared with a set of matched practices in the sentinel network and the rest of the network. We will describe any difference in implementation models in terms of staff involved and workflow.

**Conclusions:**

This study will generate data on the impact of POCT testing for influenza in primary care as well as help to inform about the feasibility of incorporating POCT into primary care workflows. It will inform the design of future larger studies about the effectiveness and cost-effectiveness of POCT to improve antimicrobial stewardship and any impact on severe outcomes.

**International Registered Report Identifier (IRRID):**

DERR1-10.2196/46938

## Introduction

### Rapid Testing for Influenza in a Postpandemic Health Service

Measures to limit SARS-CoV-2 transmission during the COVID-19 pandemic coincided with a marked decrease in infections caused by other respiratory viruses [1,2].

After the lifting of COVID-19 containment measures, the robust return of influenza, seasonal coronaviruses, parainfluenza virus, and respiratory syncytial virus (RSV) was observed in many countries including the United Kingdom [3].

**Figure 1:**
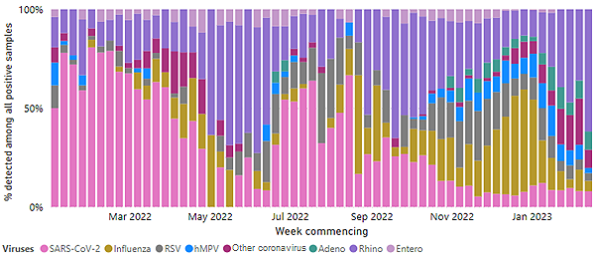
Swab positivity by week in the English national sentinel surveillance network from March 2022 to January 2023 [4]. hMPV: human metapneumovirus; RSV: respiratory syncytial virus.

Figure 1 and Table 1 show data from the English national sentinel surveillance network as of the end of November 2022. Influenza and RSV were the main circulating respiratory viruses, with SARS-CoV-2 making up only 12.4% (15/121) of the total number of laboratory-confirmed cases.

In the context of a postpandemic health care service where there are high levels of circulating influenza, accurate, rapid molecular diagnostic test platforms have the potential to (1) improve clinical decision-making regarding the use of antibiotics and antivirals, (2) improve patient outcomes due to the early appropriate use of antivirals, and (3) provide better information to inform sentinel surveillance and clinical research including studies of vaccine effectiveness and real-world trials [5,6].

Specifically, for patients with influenza infection, early diagnosis and administration of antivirals may improve clinical outcomes [7,8]. They may also limit symptom duration and spread to household contacts, and newer antivirals for influenza, such as baloxavir, have been shown to improve the time to resolution of symptoms and reduce complications in high-risk patients [9].

Currently, only a small proportion of patients with respiratory tract infections (RTIs) undergoes diagnostic microbiological testing before receiving treatments in primary care [10], and there is evidence of widespread variations in antimicrobial prescribing practices [11]. This is important, as prescribing in primary care accounts for about 8% of National Health Service (NHS) expenditure, which is equivalent to over £9 billion (US $11.1 billion) per year, with just over £220 million (US $271.5 million) being spent on antimicrobials [12]. Inappropriate prescribing of antimicrobials and unwarranted variation in prescribing can contribute to increase of antimicrobial-resistant strains and patient adverse events in the short and long term [13].

We have previously shown that, in a prepandemic context, it is feasible to undertake point-of-care testing (POCT) for influenza in primary care in the England, with promising impacts on antimicrobial use and estimates of influenza vaccine effectiveness that are comparable with published data [14-16]. However, its impact on more severe outcomes such as hospitalization and mortality following infection has not been reported.

Recent work on in-pandemic prescribing of azithromycin and doxycycline for RTIs across the sentinel network has shown scope for measures to improve antibiotic stewardship in primary care [17].

With the ending of widespread national testing for SARS-CoV-2 and other RTIs in the United Kingdom in March 2022 [18] and with high levels of circulating influenza in a post-COVID-19 health service during the autumn 2022 compared with 2021, there is a need to revisit questions about the feasibility of implementing rapid diagnosis of influenza during the expected peak of viral circulation this winter (2022-2023) season and its impact on clinical management in terms of improved antimicrobial stewardship and severe outcomes (hospitalization and mortality).

**Table 1:** Swab positivity by week in the English national sentinel surveillance network between September 2022 and November 2022 (n=818).

Week commencing	Swab positivity					
	Adenovirus (n=54)	hMPV^a^ (n=85)	Influenza (n=215)	Other coronaviruses (n=13)	RSV^b^ (n=255)	SARS-CoV-2 (n=196)
9/26/2022 (n=26), n (%)	0 (0)	0 (0)	0 (0)	0 (0)	11 (42.3)	15 (57.7)
10/3/2022 (n=54), n (%)	0 (0)	1 (1.9)	20 (37)	0 (0)	6 (11.1)	27 (50)
10/10/2022 (n=52), n (%)	0 (0)	2 (3.8)	14 (26.9)	1 (1.9)	6 (11.5)	29 (55.8)
10/17/2022 (n=70), n (%)	0 (0)	6 (8.6)	19 (27.1	2 (2.9)	17 (24.3	26 (37.1)
10/24/2022 (n=82), n (%)	0 (0)	6 (7.3)	28 (34.1	0 (0)	30 (36.6	18 (22)
10/31/2022 (n=94), n (%)	9 (9.6)	9 (9.6)	31 (33)	1 (1.1)	24 (25.5	20 (21.3)
11/7/2022 (n=95), n (%)	12 (12.6)	11 (11.6)	16 (16.8)	4 (4.2)	35 (36.8)	17 (17.9)
11/14/2022 (n=122), n (%)	6 (4.9)	18 (14.8)	34 (27.9)	2 (1.6)	42 (34.4)	20 (16.4)
11/21/2022 (n=102), n (%)	12 (11.8)	14 (13.7)	27 (26.5)	0 (0)	40 (39.2)	9 (8.8)
11/28/2022 (n=121), n (%)	15 (12.4)	18 (14.9)	26 (21.5)	3 (2.5)	44 (36.4)	15 (12.4)

^a^hMPV: human metapneumovirus.

^b^RSV: respiratory syncytial virus.

### Objectives

This study aims to report the impact of POCT for influenza on antimicrobial prescribing in primary care. We will additionally describe severe outcomes postinfection (hospitalization and mortality) and how POCT is integrated into primary care workflows.

## Methods

### Study Setting and Population

The impact of POCT for influenza on antimicrobial stewardship (PIAMS) study will take place between December 2022 and May 2023, during the influenza season as defined by the UK Health Security Agency (UKHSA). It is nested within the English national sentinel network managed by the Oxford-Royal College of General Practitioners (RCGP) Research and Surveillance Centre (RSC) at the Nuffield Department of Primary Care, University of Oxford. This network of over 1800 primary care practices is the primary infectious disease surveillance network for England, which is generally representative of the English population [19]. It has been providing weekly infection data for over 50 years that are used to monitor trends in infectious disease and investigate real-world vaccine and treatment effectiveness [20].

All practices that contribute data to the English national sentinel network will be invited to participate in the PIAMS study, and 10 practices will be selected to take part in the PIAMS study. We will prioritize practices within the network with capacity to undertake influenza POCT and who had previously been involved in SARS-CoV-2 POCT through the RAPTOR-C19 study [21]. Those practices that have a history of less than 80% complete data returns during the previous winter season will be excluded.

Each participant practice will receive training about the study, including hands-on training on how to administer a swab test and how to use the POCT analyzers.

### Case Definition of Eligible Patients

All patients registered within the PIAMS study practice who do not have an opt-out code on their medical record and are showing symptoms of influenza-like illness (ILI), acute respiratory illness (ARI), or fever of higher than 37.5 °C will be eligible for the study if they consent to participate. We will use the European Centre for Disease Prevention and Control case definitions of ILI and ARI for this study. We will use the following exclusion criteria: (1) patient has an opt-out code on their medical record or (2) no informed consent.

### Face-to-face Recruitment of Eligible Patients and Point-of-care Testing for Influenza

We will undertake opportunistic swab sampling for this study, with potential participants being identified from those registered patients who present to the study practice with respiratory symptoms described in the case definition. No screening nor eligibility assessment will be undertaken. Blinding of participants and researchers will not be undertaken.

Eligible patients or their parent or legal guardian will be approached by a practice general practitioner (GP) or research nurse to explain the study and ask for consent to take part when they present for a face-to-face consultation at the practice.

After obtaining consent, a nasopharyngeal swab will be taken by a suitably qualified and experienced GP or research nurse. For those who do not attend the practice in person, a self-test kit will be sent to their home.

The cobas Liat POCT analyzer, manufactured by Roche Diagnostics International, will be used for this study [22]. This POCT analyzer is an automated multiplex polymerase chain reaction system, with previous studies demonstrating excellent performance comparable with gold standard laboratory assays, with sensitivity and specificity in the region of 100% and 97.1%-100%, respectively, for influenza A and 97.8%-100% and 99.5%-99.7%, respectively, for influenza B when fresh, prospectively collected samples are tested [23,24]. It has CE-marking in the European Union. In the United States, it is Food and Drug Administration–cleared, and it is also Clinical Laboratory Improvement Amendments–waived for use for rapid influenza testing. This study will not assess the accuracy of the POCT analyzer but will use the machine for its approved purpose.

The swab will be inoculated in a test kit and tested on the cobas Liat analyzer as soon as possible after being taken. The results will be available to the clinician in less than 20 minutes.

Antimicrobial prescribing and other study outcomes for those who have been swabbed will be obtained by linking information from the cobas Liat analyzer with data from the patient’s computerized medical record in primary care. We will demonstrate the feasibility of reporting antimicrobial stewardship and severe outcomes postinfection. Severe outcomes postinfection such as hospitalization and mortality will be obtained by linking the patient’s primary care computerized medical record to secondary care records at the national level including hospitalization and mortality data. A recent study used a linked cohort to study severe outcomes from COVID-19 infection [25].

### Virtual Recruitment of Eligible Patients and Home Swabbing of Recruited Patients

We envisage that the majority of eligible patients will be seen face-to-face for this study. Where the eligible patient is not able to attend the practice in person, they will be told about the study remotely, and verbal consent will be taken.

A home swabbing kit will be sent to consented patients who are not able to attend the practice in person. This will include a nasal swab and instructions on how to take a self-test. Further details of our patients self-swabbing at home service have been previously published [26]. Once the nasal swab has been taken, the patient will be asked to place the swab in the included transport container with the manufacturer-approved universal transport media or viral transport media and inform the study practice staff who will arrange collection of the swab sample within 4 hours of being taken for immediate POCT.

### Clinical Workflow Integration

Data regarding the utility of the influenza POCT in primary care and the issues and barriers to implementation of influenza POCT in primary care workflows will be collected by ethnographic observation of study practices and by a semistructured survey of clinicians and practice staff at the participating practices. Information will be collected about the following domains previously found to be important to implementation of POCT sampling [27]: usability of the POCT platform, clinical pathways and training, result reporting, clinical governance, costs, monitoring of effectiveness.

The ethnographic observations, semistructured survey, and reported POCT results will be used to assess which practices were more successful at integrating POCTs into their practice processes by comparing business process models of the practices. Business process models are graphical representations of business-oriented processes within an organization. This is helpful to model collaborations and business transactions within health systems. Business processes are typically modeled using Business Process Modeling Notation (BPMN). BPMN can be used to depict the end-to-end flow of a business process. The notation has been specifically designed to coordinate the sequence of processes and the messages that flow between different process participants in a related set of business activities [28,29].

### Sample Size Calculation

The primary outcome measure is the proportion of consultations for RTI with all antibiotics prescribed during the winter season. We aim to detect a difference of ≤7% in the proportion of consultations for RTI in which antibiotics were prescribed, which is what we found previously between practices using POCT and the rest of the RCGP network [14,15]. We have assumed that the coefficient of variation between practices within the sentinel network, for the proportion of consultations with antibiotics prescribed, is 0.23 from Ashworth et al [11] with an α of .05 and a power of 80%. To detect a 5% difference in the proportion of consultations at which antibiotics are prescribed, we would need to sample 1023 people with ILI or ARI.

Previous swabbing rates across the network have averaged 6 swabs per practice per week in previous years. Given 10 practices swabbing a maximum of 3 swabs per week for 36 weeks, we would expect to collect up to 1000 face-to-face nasopharyngeal swabs from eligible people with ILI or ARI over the course of the study.

### Statistical Methods

We will undertake descriptive analysis of the swabbing rates and swab positivity rates from the study practices. This includes analysis of the demographic and clinical characteristics of those presenting with RTI symptoms and those with confirmed influenza infections in primary care. We will also report the number of patients who have also undertaken swabbing for reference laboratory analysis in addition to POCT testing in study practices. In those study practices that also take part in virological surveillance, these reference laboratory swabs are reported to the UKHSA as part of national disease surveillance.

To answer our primary objective, we will calculate the crude odds of antimicrobial prescribing (all antibiotics and all antivirals regardless of type), given a POCT diagnosis of influenza is the probability of antimicrobial prescribing given a particular POCT test result. We will record whether the antimicrobials were given within 7 days of the POCT diagnosis or within 30 days of diagnosis. We will calculate the crude odds ratio by dividing the odds of antimicrobial prescribing in people who receive a positive influenza POCT result by the odds in people who receive a negative test result.

We will also calculate the adjusted odds ratio using a logistic regression model. We will use the following set of covariates that are known to be associated with the severity of influenza and are collected from the routine data extracted from the electronic health record: age, sex, ethnicity (reported in 5 categories: White, Asian, Black, other, or mixed; maximized using an ontology [30]), socioeconomic status (measured using the index of multiple deprivation [31], which is a nationally available measure assigned based on post code), any of these chronic underlying conditions (chronic pulmonary disease, cardiovascular disease, diabetes, liver disease, renal disease, neurologic or neuromuscular conditions, treatment-induced immunosuppression, and disease-induced immunosuppression), number of GP visits in the 12 months prior to the study period describing a study participant’s health care–seeking behavior, number of hospitalizations in the 12 months prior to the study period (used as proxy for the severity of the chronic conditions), influenza vaccination in previous influenza seasons (at least one), pregnancy, use of influenza antivirals, and pneumococcal and COVID-19 vaccination status.

To answer our secondary objective, we will select a matched set of practices from the rest of the English national sentinel network based on practice size, average age of registered patients, proportion of registered female patients, and number of GPs employed during the study.

The rates of hospital referrals and deaths related to influenza infection within 30 days of diagnosis will be compared between PIAMS study practices and the rest of the network using the ANOVA test.

### Ethical Considerations

The study was approved by the English National Research Ethics Committees (Integrated Research Application System reference: 292961; REC reference: 21/YH/0077).

A practice GP or research nurse will undertake informed consent. This person will be suitably qualified and experienced and have been authorized to do so by the principal investigator. Once an eligible patient is identified, they or their parent or legal guardian will be approached by a practice GP or research nurse. Written and verbal versions of the participant information and informed consent will be presented to the patient or their parent or legal guardian detailing no less than the exact nature of the study, what it will involve for the participant, the implications and constraints of the protocol, and the known side effects and any risks involved in taking part. It will also be clearly stated that the participant is free to withdraw from the study at any time for any reason without prejudice to future care, without affecting their legal rights, and with no obligation to give a reason for withdrawal.

The patient will be allowed as much time as wished to consider the information and provided the opportunity to question the investigator, their GP, or other independent parties to decide whether they will participate in the study. Written informed consent will then be obtained by means of a dated signature and the dated signature of the person who presented and obtained the informed consent. A copy of the signed informed consent will be given to the participant. The original signed form will be retained at the study site.

When the eligible patient is not able to attend the practice in person, they will be told about the study remotely by a trained GP or nurse. The trained GP or nurse will go through the remote consent form with the participant over the phone. Once the participant has provided verbal consent to take part in the study, the researcher will sign a copy of the remote consent form and will provide the participant with a copy via a secure method such as NHS email. The patient will then be sent a swab to take at home and return to the practice within 4 hours of being taken for immediate POCT. Collection of the sample will be arranged by study staff.

For the ethnographic observations, we will verbally inform and orally consent the clinicians, a PIAMS researcher will record the consent process by completing a Record of Consent Form and the form will be signed in the presence of the participant to confirm oral consent. We will also provide information about the PIAMS study on practice posters to notify the patients about the observation activities associated with the nested qualitative study. Their consent will be implied by not opting out. A PIAMS researcher will be available to answer any questions they may have about observations undertaken for the nested qualitative study. Both clinicians and patients have the right to withdraw from participation or to decline to participate at any time without reason.

For the semistructured questionnaire, all survey respondents will complete an informed consent question embedded at the beginning of the anonymous questionnaire. If the participants answer “YES” to the first question of the form, this will imply their consent to participate, and the survey will begin. If the participants answer “NO” to the informed consent question, they will directly exit the current page. No participant will be forced to participate in the survey, and their participation will be based on their agreement, which can be withdrawn at any time.

Pseudonymized information about swab samples from consented patients, including participant ID, site ID, and swab result, will be stored in the POCT machines. These data will be extracted weekly from the machines and sent via an encrypted email to the study team to be stored in the research group’s secure network.

Data from study practice medical records will be extracted twice weekly from information systems of the RCGP RSC practices by Apollo, part of Wellbeing Software. These records are pseudonymized as close to the source as possible by Apollo using a nonreversible “hash” algorithm. Patients who decline to share their data are excluded from the extraction process.

Data for the study are held on dedicated secure servers at the RCGP data and analytics hub in the Clinical Informatics and Health Outcomes Research Group, University of Oxford. The research group’s secure network is located behind a firewall within the university’s network; all in-bounded connections are blocked, but out-bounded connections are allowed. Only staff members or associated members of the research group approved by the head of department can access the data from secure workstations or secure laptops with encrypted drive. All staff members of the research group working within the team base work from secure workstations or secure laptops with encrypted drive within the research group’s secure network. The use of personal equipment is not permitted and cannot be connected to the secure network. A risk assessment of the physical security of the research group's offices and server room has been conducted by the building and facilities manager, the faculty information technology (IT) service manager, and the research group's information governance lead.

Survey data for the nested qualitative substudy are securely collected using Jisc (online surveys). The university has a license agreement to use this service and has recommended the service for gathering confidential data. Extraction of the survey data is only available to people with a registered Jisc account, which the university has to approve. Only authorized study researchers will be able to export survey data once logged in to Jisc. Once survey data are extracted, the data will be stored within the secure Oxford-RCGP Clinical Informatics Digital Hub (ORCHID) network. Access to your data is strictly controlled. Only authorized study researchers will have access to only pseudonymized research data from within the aforementioned secure ORCHID network.

The university is compliant with the Data Protection Act and UK GDPR and has systems for technical and organizational controls for information security, including a university-level Information Security and Governance Group chaired by the university senior information risk owner. The research group’s private network has its own system-level security policy and is tested for vulnerabilities annually.

Study practices are provided with a fee for participating in this study to cover the costs of training staff and hosting the study. A small remuneration is also provided to practices for each swab taken to cover the additional time taken during each consultation to undertake swabbing for this study. Patients are not remunerated for taking part in this study.

## Results

The study started in December 2022, and we expect to complete the final analysis for the study by June 2023.

### Baseline Data From the English National Sentinel Surveillance Network

In terms of context for this study, currently, the English national sentinel network had grown from just over 500 practices in 2019 to 1879 practices by October 2021. This represents 28.6% of English primary care practices and 31% of the population (N=17,560,196). The sentinel network’s population was found to be broadly representative of the national population in terms of age, gender, ethnicity, NHS Region, socioeconomic status, obesity, and smoking habit [19].

Latest data from across the sentinel network, between October 2020 and October 2021, show that practices have collected >8000 virology swab samples for reference laboratory testing [19]. Figure 1 and Table 1 show the latest virology swab positivity by week in the English national sentinel surveillance network prior to the start of the study.

### Protocol Amendments

Important protocol amendments will be referred to the English National Research Ethics Committees for ethical approval. Once approved, changes will be communicated directly with the recruiting study practices. The amended protocol will be shared with all relevant parties (eg, investigators and clinical research networks) in a timely manner.

## Discussion

### Overview

This study will generate data on the impact of POCT testing for influenza in primary care, especially information on its impact on antimicrobial prescribing. Additionally, the study will describe severe outcomes postinfection (hospitalization and mortality) and how POCT is integrated into primary care workflows.

It will be a chance to revisit questions about the feasibility of implementing rapid diagnosis of influenza following the end of widespread national testing for SARS-CoV-2 and other RTIs in the United Kingdom and previous studies about its promising impacts prepandemic [14-16].

### Limitations

The nonrandomized design is a limitation of our study. However, we feel that there is still value in examining the objectives within this study using a mixed methods approach as there are many questions about the challenges and barriers to implementation of rapid diagnostic testing in primary care, including the variability of adoption in different practices that are important to understand [15]. Unpacking these factors using a mixed methods design will be important to design a successful experimental study at scale to answer questions related to health outcomes. Additionally, information about influenza subtype is not available from the POCT machines used in this study and will restrict interpretation of our results, especially for calculating influenza vaccine effectiveness. A possible solution to this would have been to ask study practices to send their swabs to the national reference laboratory for additional analysis. However, this would have introduced a further step in the testing procedure in the clinic, which may have increased the risk of aerosol generation. Given that the study was originally proposed during the COVID-19 pandemic, we felt it was important to minimize the risk of generating aerosol droplets and thus did not include this in the study.

### Strengths

A strength of the study is that it is conducted among practices that are nested in the RCGP RSC English sentinel surveillance network and had also been involved in POCT sampling in previous years. This ensured that practice staff had experience with using rapid diagnostic machines, minimizing the number of spoiled samples. It will also allow comparisons of the performance of practices using POCT for influenza testing with other practices in the sentinel network that participate in the usual virology sampling program conducted by the UKHSA.

### Conclusion

Building on previous work showing the promising impacts of rapid, near-patient testing on antimicrobial use in primary care within the context of a prepandemic health service, this study will help inform about the feasibility of incorporating POCT into primary care workflows. It will inform the design of future larger studies about the effectiveness and cost-effectiveness of POCT to improve antimicrobial stewardship and any impact on severe outcomes.

## Abbreviations

ARI: acute respiratory illness
BPMN: Business Process Modeling Notation
GP: general practitioner
ILI: influenza-like illness
NHS: National Health Service
ORCHID: Oxford-Royal College of General Practitioners Clinical Informatics Digital Hub
PIAMS: impact of POCT for influenza on antimicrobial stewardship
POCT: point-of-care testing
RCGP: Royal College of General Practitioners
RSC: Research and Surveillance Centre
RSV: respiratory syncytial virus
RTI: respiratory tract infection
UKHSA: UK Health Security Agency

## Data Availability

Data cannot be shared publicly because the data are owned by the Oxford Royal College of General Practitioners (RCGP) Research and Surveillance Centre (RSC) and its participating practices. Data are available from the University of Oxford Institutional Data Access Committee (contact via ku.ca.xo.chp@nanigsuled.nomis) for researchers who meet the criteria for access to confidential data. The data underlying the results presented in the study are available from [32]. Further enquiries about the RCGP RSC network and data requests can be found at [33] or by emailing ku.ca.xo.chp@seiriuqneecitcarp.
